# PPAR**γ** and Oxidative Stress: Con(**β**) Catenating NRF2 and FOXO

**DOI:** 10.1155/2012/641087

**Published:** 2012-03-05

**Authors:** Simone Polvani, Mirko Tarocchi, Andrea Galli

**Affiliations:** Gastroenterology Unit, Department of Clinical Pathophysiology, University of Florence, Viale Pieraccini 6, 50139 Firenze, Italy

## Abstract

Peroxisome-proliferator activator receptor *γ* (PPAR*γ*) is a nuclear receptor of central importance in energy homeostasis and inflammation. Recent experimental pieces of evidence demonstrate that PPAR*γ* is implicated in the oxidative stress response, an imbalance between antithetic prooxidation and antioxidation forces that may lead the cell to apoptotic or necrotic death. In this delicate and intricate game of equilibrium, PPAR*γ* stands out as a central player devoted to the quenching and containment of the damage and to foster cell survival. However, PPAR*γ* does not act alone: indeed the nuclear receptor is at the point of interconnection of various pathways, such as the nuclear factor erythroid 2-related factor 2 (NRF2), Wnt/*β*-catenin, and forkhead box proteins O (FOXO) pathways. Here we reviewed the role of PPAR*γ* in response to oxidative stress and its interaction with other signaling pathways implicated in this process, an interaction that emerged as a potential new therapeutic target for several oxidative-related diseases.

## 1. Introduction

### 1.1. On Reactive Oxygen Species and Oxidative Stress

Reactive oxygen species (ROS) are free radicals and reactive metabolites containing oxygen with unpaired electron; potentially harmful, they act as potent oxidants for lipids, proteins, and DNA [1]. Small amount of ROS are normally produced by mitochondrial respiratory chain during metabolic reactions [2]; however they are not unnecessary and unavoidable byproducts of the respiratory chains. In fact, it is fairly accepted that the cells may produce ROS intentionally and that ROS play an important role in cellular processes such as cell-cycle progression, regulation of signaling pathways in response to intra- and extracellular stimuli, and inflammation [3–5].

Because high levels of ROS may be detrimental, the cells possess a vast array of antioxidant systems devoted to ROS neutralization and maintaining of a balance between prooxidants and antioxidants. We distinguish two types of antioxidants: enzymatic and nonenzymatic (or chemical). Enzymatic antioxidants are proteins such as superoxide dismutase (SOD), glutathione peroxidase (GPx), glutathione reductase, and catalase, while chemical antioxidants are scavenger molecules such as vitamin C and D and glutathione (GSH) [6]. Prooxidants are enzymes like NADPH oxidase, cyclooxygenase 2 (COX-2), and inducible nitric oxide synthase (iNOS) [7–9].

When the near equilibrium condition between prooxidants and antioxidants is perturbed, the ensuing imbalance determines the so-called oxidative stress. During oxidative stress the sustained production of ROS and of other high reactive species, not sufficiently quenched by antioxidants, may deal significant damage to the cells, and, if too much damage is done, that may lead to necrotic or apoptotic cell death.

Excessive ROS production and oxidative stress is believed to be the cause, or is linked to, the establishment of several pathologies (for a list see [6]), including alcoholic and nonalcoholic fatty liver disease (NAFLD) [10, 11]. ROS production and oxidative stress are also associated with the pathogenesis of HIV, HCV, and HBV viral infections [12–14].

### 1.2. PPAR*γ*

The Peroxisome-proliferator activator receptors (PPARs) form a family of ligand-activated nuclear receptor transcription factors that regulates the function and expression of complex gene networks, especially involved in energy homeostasis and inflammation [15–17]. The family comprises three known members: PPAR*α*, PPAR*γ*, and PPAR *β*/*δ*, also known as NR1C1, NR1C3, and NR1C2 [18]. Among the PPARs, PPAR*γ* is the only receptor known to possess three splicing isoforms characterized by a different tissue distribution [15, 16].

PPAR*γ* shares a protein structure common to the other PPARs and to most of the nuclear receptors characterized by 4 functional domains (called from the N terminal to the C terminal, A/B, C, D, and E/F) [15, 19]. The N-terminal region, corresponding to the A/B domain, contains the AF-1 domain (implicated in ligand-independent activation); the highly conserved DNA-binding domain, located in the C domain, is separated by the flexible hinge domain of the D region (to whom dock coactivators) from the moderately conserved ligand-binding domain (E/F domain) that contains also the AF-2 region for ligand-dependent activation that is also essential for coactivators recruitment [15, 16, 19].

PPAR*γ* does not act alone but regulates genes transcription acting as heterodimer with the retinoid X receptor (RXR) [15, 16]. As dimers, PPAR*γ* : RXR bind to PPAR response elements (PPREs) located in the promoter region of target genes. PPREs are hexanucleotide direct repeat consensus elements, whose sequence is AGGTCA, separated by one or two bases (known as Direct Repeat 1 (DR-1) and 2 (DR-2)).

The activity of PPAR*γ* in a cell context depends on the presence of other molecules such as coactivators and corepressors, expression of other PPARs, availability of RXR, the status of promoter of the target genes, and presence of endogenous ligands [20]. Activation of PPAR*γ* is canonically obtained through ligand binding. The ligand-binding determines a conformational change in the three-dimensional structure of PPAR*γ* : RXR dimers that is accompanied by loss of heat shock proteins, detachment of corepressors, and recruitment of coactivators [15, 16, 21]. Changes in nuclear localization of PPAR*γ* have also been described [22]. Akiyama et al. [22] utilizing fluorescent-tagged PPAR*γ* and RXR demonstrated that PPAR*γ* nuclear distribution changes after the coexpression of RXR independently from the presence or absence of PPAR*γ*- or RXR-specific ligands, suggesting that RXR is implicated in the nuclear reorganization of PPAR*γ*.

The activation status of PPAR*γ* may also be modulated by posttranslational modifications, such as phosphorylations, independently from ligand binding [21, 23]. Indeed, the mitogen-activated protein kinase(s) (MAPK) p38, extracellular-signal-regulated kinase (ERK), and c-Jun N-terminal kinases (JNKs) are able to phosphorylate PPAR*γ* [21]; this event is usually associate with inhibition of transcriptional activity [21], cytoplasmic localization [24, 25], and possibly degradation [26] of PPAR*γ*. The activated receptor then binds to PPRE: the net results of this complex mechanism are changes in the expression level of mRNAs encoding PPAR*γ* target genes.

PPAR*γ* may also influence genes expression with a mechanism known as transrepression. Transrepression does not require DNA binding of the activated receptor to the PPRE and has been invoked to explain anti-inflammatory action of PPAR*γ* [27–30]; several models to explain the transrepression have been proposed including competition for coactivators molecules, for co-receptors, or for the binding sites [31].

The experimental evidence that oxidized lipids and 15-deoxy-delta (12, 14)-prostaglandin J(2) (15d-PGJ2), which are natural endogenous PPAR*γ* ligands [32], are produced during oxidative stress and inflammation suggests that PPAR*γ* is implicated in oxidative stress response. This hypothesis is also corroborated by the sheer number of scientific papers published on this topic: in fact, a query for “PPAR*γ* and oxidative stress” in PubMed returns (as to August 2011) at least 300 publications.

Given that various pathologies are associated to oxidative stress and PPAR*γ* is emerging as an important regulator of the response to this condition, in this paper we will summarize the current understanding of the role of this nuclear receptor in response to oxidative stress and its interaction with other signaling pathways implicated in this process.

## 2. PPAR**γ** and Oxidative Stress Response

### 2.1. PPAR*γ*: An Emerging Anti-Inflammatory and Antioxidant Gene

The insulin-sensitizing drugs thiazolidinediones (TZDs), used in the treatment of type II diabetes, are PPAR*γ*-specific agonists. Together with other PPAR*γ* agonists, they are used or are under studies for the treatment of oxidative stress-related diseases such as diabetes, vascular diseases [33–35], Parkinson's [36], Alzheimer's [37], nonalcoholic steatohepatitis (NASH) [38], and Huntington's [39]. It is worth noting that although for a long time TZD effects have been thought to be PPAR*γ* dependent, TZD PPAR*γ*-independent effects have been widely described and may coexist with the PPAR*γ*-dependent effects [40–44]. As examples, TZDs have anticancer activity in the liver, independently of PPAR*γ*, via inhibition of nucleophosmin that is associated with the induction of p53 phosphorylation and p21 expression [40]; in QZG hepatocytes rosiglitazone, independently of PPAR*γ*, decreases ROS generation induced by high glucose treatment, whereas it induces the antioxidant enzyme heme-oxygenase 1 (HO-1) in a PPAR*γ*-dependent manner [41].

**Figure 1:**
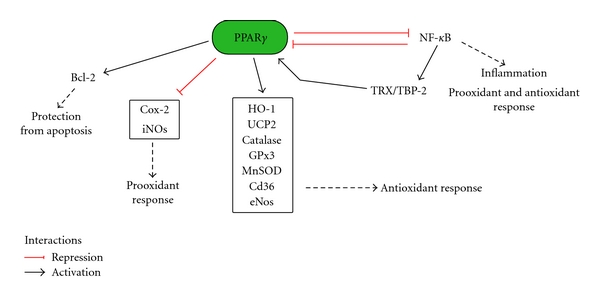
PPAR*γ* target genes and cellular roles. In oxidative stress conditions the nuclear receptor PPAR*γ* directly regulates a vast array of genes involved in the response to oxidative stress and exerts anti-inflammatory effects transrepressing NF-*κ*B.

Nonetheless, PPAR*γ* may directly modulate the expression of several antioxidant and prooxidant genes in response to oxidative stress (Figure 1). The mouse, human, and rat catalase [45, 46], a major antioxidant enzyme that decomposes H_2_O_2_ to H_2_O and O_2_, is transcriptional regulated by PPAR*γ* through PPREs containing the canonical DR-1 [46] and that may be located as far as 12 kb from the transcription initiation site [45]. Interestingly, H_2_O_2_ induces apoptosis in cardiomyocytes [47] through a marked downregulation of the antiapoptotic protein B-cell lymphoma 2 (Bcl-2) and may reduce PPAR*γ* expression in HUVEC cells, an effect that is reverted by catalase [48]. Besides, PPAR*γ* protects cardiomyocytes and glial cells from oxidative stress-induced apoptosis inducing Bcl-2 [47, 49]. This prosurvival action of PPAR*γ* is probably independent of the MAPKs and AKT pathways [47, 49].

Furthermore, ligand-activated PPAR*γ* promotes the expression of manganese SOD (MnSOD) [50, 51], GPx3 [52], the scavenger receptor CD36 [53, 54], endothelial nitric oxide synthase (eNOS) [33], HO-1 [41, 55, 56], and the mitochondrial uncoupling protein 2 (UCP2) [35, 38], whereas it downregulates COX-2 and iNOS [29, 57–59].

The activity of mitochondrial MnSOD, which oversees the dismutation of O_2_^−^ to O_2_ and H_2_O_2_, is increased by rosiglitazone [50]; moreover in PPAR*γ* knockout mice MnSOD is downregulated at the transcriptional and translational levels with a consequent increase of O_2_^−^ levels [51]. In fact, promoter analysis revealed that MnSOD is a direct target of PPAR*γ* [51].

GPx protect cells from oxidative stress in two ways, reducing H_2_O_2_ to H_2_O and O_2_ (like the previously cited catalase) and acting as scavenging for oxidized lipids. Recently, Chung et al. [52] demonstrated that in human skeletal muscle cells TZD-mediated activation of PPAR*γ* induces GPx3 and protects from oxidative stress.

In spontaneous hypertensive rats, oral intake of rosiglitazone upregulates UCP2 and the protective effects of rosiglitazone are abrogated silencing PPAR*γ* [35]. The mitochondrial UCP2 may protect from oxidative stress preventing the accumulation of O_2_^−^ in the mitochondria and facilitating the transport of mitochondrial ROS to the cytosol, where they would stimulate the expression of neuroprotective genes (e.g., MnSOD and Bcl-2) [60].

The expression of the scavenger receptor CD36 that mediates the recognition and internalization of oxidized lipids [61] may also be regulated by PPAR*γ*. In fact, it has been demonstrated that in murine macrophages CD36 expression increases after the treatment with PPAR*γ* ligands [54], probably as a consequence of PPAR*γ* binding to functional active PPRE located in the gene promoter [53].

PPAR*γ* modulates the expression of eNOS and iNOS [29, 33, 59]. These enzymes produce NO from arginine; when NO is produced in high quantities it may react with O_2_^−^ forming the highly reactive peroxynitrite. In mice with an endothelial specific knockout of PPAR*γ* aortic segments release less nitric oxide than those from controls and the reduced expression correlates with an increase in the parameters of oxidative stress [33], suggesting that PPAR*γ* protect from oxidative stress controlling eNOS expression. Conversely, the high production of NO by iNOS is usually associated to complex immunomodulatory and antitumoral mechanisms and dysfunctional induction of iNOS expression seems to be involved in the pathophysiology of several human diseases [9]. PPAR*γ* agonists repress iNOS expression in various cells such as activated macrophages [59], lipotoxic pancreatic islets [29], and lipopolysaccharide-(LPS-) activated Schwann cells [62] suggesting a protective role of PPAR*γ* from reactive peroxynitrite.

COX-2 is an inducible form of ciclooxygenase that contributes to the metabolism of arachidonic acid forming prostaglandin H2 (PGH2), a precursor of 15d-PGJ2 [7, 8]. The production of PGH2 requires the presence of free radicals and may also produce O_2_^−^, contributing to oxidative stress. Besides, PGH2 also inhibits apoptosis, favors cell adhesion, motility, invasion, and promotes angiogenesis [8]. How PPAR*γ* acts on COX-2 expression is still debated. In fact during oxidative stress a PPAR*γ*-mediated inhibition as well as induction of COX-2 has been reported [41, 57, 58]. Collino et al. [58] and Zhao et al. [57] reported that the PPAR*γ* agonist pioglitazone and rosiglitazone protect the cells from oxidative stress after ischemia suppressing COX-2 expression in a PPAR*γ*-dependent manner [57]; conversely, Wang et al. [41] demonstrated that high glucose significantly reduced the expression of COX-2 and rosiglitazone upregulated COX-2 expression in a PPAR*γ*-dependent manner. This latter result is in agreement with that of Aleshin et al. [63] that described a TZDs enhancement of LPS-induced COX-2 expression via PPAR*γ*-dependent pathway.

Still debated is also the effect of PPAR*γ* on the expression of HO-1. HO-1 can be strongly induced in many tissues in response to cellular stress caused by a wide spectrum of *stimuli*, including ROS and prostaglandins [64]. Enzymatic activity of HO-1 reduces oxidative stress, diminishes inflammatory response, and lowers the rate of apoptosis [64]. In human vascular cells PPAR*γ* induces HO-1 expression interacting with two PPRE DR-1 located between −1740 kb and −1826 kb from the transcription start site [55]. Moreover PPAR*γ* increases the expression of HO-1 during oxidative stress induced by high glucose [41] and in the age-related macular degeneration [56]; however rosiglitazone decreases the expression of HO-1 in the hippocampus after the status epilepticus [50]. To further complicate the subject, PPAR*γ* agonist 15d-PGJ2 may induce HO-1 independently of PPAR*γ* *via* nuclear factor erythroid 2-related factor 2 (NRF2) [65] or GSH-dependent mechanisms [42].

PPAR*γ* may also influence the antioxidant and anti-inflammatory responses interacting with other regulatory pathways. PPAR*γ* has shown to induce anti-inflammatory responses inhibiting proinflammatory transcription factors such as nuclear factor Kappa-light-chain-enhancer of activated B cells (NF-*κ*B) [28–30, 66] (Figure 1). The NF-*κ*B factors are of central importance in inflammation and their role in response to ROS has been recently reviewed [7]. NF-*κ*B action is usually proinflammatory and prooxidant inducing the expression of genes such as iNOS and COX-2, but it may also regulate the expression of SOD and other anti-inflammatory genes [7]. PPAR*γ* may reduce NF-*κ*B activities in various ways. PPAR*γ* transrepresses NF-*κ*B activation either by forming a repressor complex in the promoter of NF-*κ*B-target genes or by direct binding with NF-*κ*B [28–30]; moreover the nuclear receptor may reduce NF-*κ*B activation mediated by H_2_O_2_ [66] probably through increased expression of catalase [45, 46]. Conversely, NF-*κ*B negatively regulates PPAR*γ* transcriptional activity with a mechanism that requires the presence of histone deacetylase 3 (HDAC3) [67, 68]. Interestingly, NF-*κ*B could, potentially, also induce PPAR*γ* via a thioredoxin- (TRX-) dependent mechanism. TRX is a potent antioxidant protein whose expression depends on NF-*κ*B [7]; the activity of TRX is negatively modulated by the TRX-binding protein-2 (TBP-2) [69]. TBP-2 null mice treated with a methionine-choline-deficient diet show simple steatosis but not steatohepatitis with reduced oxidative stress and increased expression of PPAR*γ*, suggesting a link between TRX-TBP-2 and the nuclear receptor [69].

Furthermore, new experimental pieces of evidence suggest that PPAR*γ* modulates oxidative stress responses interacting with NRF2 and the Wnt/*β*-catenin and forkhead box proteins O (FOXO) pathways.

### 2.2. Interacting with NRF2

The nuclear factor erythroid 2-related factor 2 (NRF2), a redox-sensitive member of the cap “n” collar basic leucine zipper family, plays a vital role in cytoprotection against oxidative and electrophilic stress as well as in suppression of inflammation [70].

Expression of NRF2 is tightly regulated by the Kelch-like ECH-associated protein 1 (Keap1) [71], an E3 ubiquitin ligase, that mediates also the downregulation of NF-*κ*B signaling by targeting the inhibitor of NF-*κ*B subunit *β* (IKK*β*) [72].

In unstressed conditions, NRF2 remains in the cytosol and it is rapidly degraded by proteasome after Keap1-mediated ubiquitination. In presence of ROS or electrophilic agents, the degradation ceases and NRF2 translocates and accumulates in the nucleus where it regulates the transcription of genes containing in the promoter region the antioxidant response element (ARE). NRF2 nuclear translocation and transcriptional activity depend also on the phosphorylation status of the transcription factor [41, 73–75]; AKT-, p38-, and protein-kinase-C-(PKC-) induced phosphorylation seems to be the principal mechanism in NRF2 activation [41, 73–75].

The number of targets of NRF2 is continuously expanding and comprises NADPH-generating enzymes [76], glutathione S-transferase (GST) [77], CD36 [54, 74], HO-1 [65, 75], and NRF2 itself [78].

Recently, microarray analysis of NRF2 target genes demonstrated that PPAR*γ* expression is compromised in NRF2 null mice, in normal and stress conditions [79–81]. In the lungs of NRF2 null mice, hyperoxia-induced expression of PPAR*γ* was markedly reduced compared to wild-type animals [79, 80], whereas in the liver of 6-month old NRF2 null mice the genes involved in lipid synthesis and uptake, including PPAR*γ*, were generally downregulated compared with the wild-type mice [81]. The PPAR*γ* reduced expression is a direct effect of the lack of NRF2; in fact both Huang et al. [81] and Cho et al. [80] demonstrated that NRF2 induces PPAR*γ* expression binding to at least two ARE sequences (−916 and −784 ARE, resp.) in the upstream promoter region of the nuclear receptor.

As previously stated, PPAR*γ* agonists exert potent anti-inflammatory activities; interestingly, rosiglitazone and other PPAR*γ* ligands such as 15d-PGJ2 increase NRF2 expression dependently from PPAR*γ* [73, 77, 82]. Furthermore, NRF2 expression is attenuated in the lung of mice with decreased PPAR*γ* levels and putative PPREs have been identified on NRF2 gene promoter [77, 80]; this suggests that a positive feedback loop exists among PPAR*γ* and NRF2, where PPAR*γ* may act directly or through upstream pathways for NRF2 activation. This circuit may not only explain the effects of PPAR*γ* agonists on NRF2 expression but may also contribute to the anti-inflammatory effects associated with PPAR*γ* activation. Thimmulappa et al. [83] demonstrated that NRF2 deficiency causes greater sensitivity to septic shock and results in disregulation of the expression of effector genes of the innate immunity that are under the control of NF-*κ*B, increasing lung inflammation. In fact, NRF2 regulates NF-*κ*B activation largely by modulating its MyD88-dependent and -independent upstream signaling components; the restrain of proinflammatory signaling pathways is apparently associated with the NRF2 ability to maintain redox homeostasis. In this sense, the PPAR*γ*-mediated activation of NRF2 signaling, which results in NRF2-dependent inhibition of proinflammatory pathways, may strengthen, and contribute to, the anti-inflammatory activity of PPAR*γ*.

The altered expression of genes involved in inflammation and lipid metabolism in NRF2 null mice is interesting for the pathogenesis of NAFLD and its progression to NASH. Lack of NRF2 in mice exposed to high-fat or methionine-choline diet increases the oxidative stress-related damage and the lipid accumulation mediated by LXR*α* [84, 85]. The pathogenesis of human NASH is usually explained by a “two-hit” model where the “first hit” is the steatosis and the second is constituted by many factors, including oxidative stress. Although there is a controversy on the relationship between high-fat diet feeding and NRF2 expression in mice (both increase and decrease of gene expression have been reported [85]), human samples from steatotic livers show a marked decrease in NRF2 expression [85]. Reduced expression or loss of NRF2 and/or PPAR*γ* may increase oxidative stress that acts as “second hit” in NASH; in fact, on the basis of experimental evidence, we may hypothesize that NRF2, together with PPAR*γ*, preserves from the progression of NASH throughout protective effects on oxidative stress, inflammation, and lipid metabolism.

What is certain is that PPAR*γ* is not a simple downstream effector of the NRF2 pathway but may also act synergically with NRF2 in the activation of antioxidant genes [54, 77]. In the GSTA2 promoter ARE and PPRE response elements coexist [77]; Park et al. [77] demonstrated that the ARE site had essential roles in the transactivation of the *GSTA2* gene by PPAR*γ* and RXR ligands, as evidenced by the binding site-deleted promoter-luciferase assay, and it is necessary for the full gene transactivation [77]. Similarly the expression of the scavenger receptor CD36 in murine macrophages is dependent on both proteins [53, 54]. On the other hand, the case of the CD36 is also explanatory of the fact that PPAR*γ* and NRF2 may act independently to induce the transcription of the same gene, depending on the cellular context. In murine macrophage, oxidized lipids that in macrophages induce a PPAR*γ*-dependent CD36 expression [53, 54], in preadipocytes induce a mostly PPAR*γ*-independent expression of the scavenger [74]; the latter is associated to increase expression and nuclear translocation of NRF2 consequently to PKC activation [74].

### 2.3. The Involvement of Wnt/*β*-Catenin Pathway and FOXO and Their Interaction with PPAR*γ*

The Wnt/*β*-catenin pathway is a well-known morphogenic signaling pathway first discovered in colon carcinoma [86]. From this very first discovery the role of Wnt and *β*-catenin has been described in several processes including morphogenesis, maintenance of stem cells characteristics but also cell differentiation, cell survival, and lipid and glucose metabolism [87–91]; in fact Wnt pathway belongs to a restricted group of extremely evolutionary conserved signaling cascades. The pathway is complex and quite versatile in terms of end effects on cellular behavior.

The main effector of Wnt, in the canonical pathway, is the bipartite transcription *β*-catenin-DNA binding protein T cell factor/lymphoid enhancer factor (TCF/LEF) [86]; Wnt may also act independently of *β*-catenin in the so-called noncanonical pathway [92, 93] and regulate proteins translation *via* mTOR [94].

*β*-catenin is constitutively expressed in the cytoplasm, but its activity is regulated at the posttranscriptional level through phosphorylation. *β*-catenin phosphorilation is performed by a degradation complex comprised, among other proteins, of glycogen synthase kinase 3 beta (GSK3*β*) and adenomatous polyposis coli (APC) and results in ubiquitination and degradation by the proteasome.

Conversely, the binding of Wnt glycoproteins to the seven pass membrane receptor Frizzled and coreceptors low-density lipoprotein receptor-related protein 5/6 (LRP5/6) results in GSK3*β* phosphorylation followed by the stabilization and nuclear translocation of *β*-catenin, and hence activation of the pathway. To add further complexity to the system, other signaling pathways, such as the insulin and insulin-like growth factor 1 (IGF-1) pathways, activate *β*-catenin possibly through an AKT-dependent GSK3*β* phosphorylation [95, 96].

Recent experimental pieces of evidence suggest that the Wnt/*β*-catenin is deeply involved in the response to oxidative stress. Products of lipid oxidation activate the canonical Wnt pathway in a rat model of diabetic retinopathy [97] and Wnt is implicated in the genesis of diabetes followed by ROS stress [91]; conversely, ethanol may reduce cellular localization of *β*-catenin and TCF/LEF gene transcription in the bone [98], while nucleoredoxin, a thioredoxin-related protein, that is inactivated by oxidative stress, blocks the pathway [99, 100]. The Raf kinase inhibitory protein (RKIP), an inhibitor of the c-Raf and NF-*κ*B, may influence GSK3*β* phosphorylation: RKIP depletion increases ROS-induced p38 MAPK activation which inhibits GSK3*β* increasing *β*-catenin levels [101]. Furthermore, ROS generation induced by high glucose destabilizes the *β*-catenin and induces cell-cycle arrest and apoptosis [102]. In the mouse, loss of *β*-catenin in the developing liver determines embryonic lethality at E17 associated with increased apoptosis and oxidative stress [103]; these results are confirmed in mouse diethylnitrosamine-(DEN-) induced liver carcinogenesis, where the absence of *β*-catenin increases the oxidative stress [104]. Interestingly, Wnt/*β*-catenin may also be involved in the regulation of antioxidant enzymes such as GST [105] and cytochrome P450 (CYP) [106].

The described experimental results not only demonstrate the involvement of the Wnt/*β*-catenin in the response to oxidative stress but also imply that its role is multifaceted. Interestingly, some of the variegated effects of the Wnt/*β*-catenin may be explained by the introduction of another player in this complex regulatory pathway: the FOXO proteins.

The FOXO subfamily of forkhead transcription factors is emerging as a fundamental regulator of *β*-catenin/TCF activation and plays an important role in stress response to ROS. In mammals, the FOXO family comprises four isoforms (FOXO1, FOXO3, FOXO4, and FOXO6) characterized by an overlapping expression during development and in adulthood. FOXO acts as transcriptional activators governing a variety of different cellular processes in response to different environmental contexts specifically promoting cell-cycle arrest, apoptosis, glucose metabolism, and stress resistance [107–109]. Kops et al. [107] and Essers et al. [109, 110] demonstrated that FOXO1, 3, and 4 are also directly involved in cell responses to ROS inducing the MnSOD; FOXO may also regulate catalase production and reduce *β*-catenin expression [110]. FOXO activation by ROS is probably mediated by the JNK MAPK pathway: in fact, oxidative stress induced by treatment of cells with H_2_O_2_ results in the activation of the small GTPase Ral that determines the phosphorylation and activation of JNK and JNK-mediated phosphorylation of FOXO1 [109]. Conversely, the phosphorylation of FOXO proteins mediated by the PI3K-AKT, p38, or IKK*β* kinases inhibits FOXO function inducing cytoplasmic translocation and protein degradation [108, 111–114].

*β*-catenin binds directly to FOXO through an evolutionary conserved mechanism and enhances FOXO transcriptional activity in mammalian cells [110]. In stress conditions FOXO proteins compete with TCF for a limited pool of available *β*-catenin, thereby inhibiting TCF transcriptional activity [115, 116]. *β*-catenin then appears to fulfill a critical function in balancing TCF signaling (mainly proliferative) and FOXO signaling (mainly devoted to stress response and apoptosis).

A cross-talk among PPAR*γ*, *β*-catenin, and FOXO is demonstrated by several studies. The existence of an influence of Wnt/*β*-catenin on PPAR*γ* expression has been reported in 2000 when Ross et al. [117] described that Wnt signaling prevented the adipogenesis by silencing PPAR*γ* expression; however, the interaction between PPAR*γ* and Wnt stretches out the adipogenesis and has been described in several processes where ROS production and oxidative stress are important such as aging, Alzheimer's and Parkinson's diseases, diabetes, hepatic stellate cells activation, liver fibrosis, and cancer [6, 118–121].

The molecular pathway linking Wnt to the repression of PPAR*γ* has been elucidated only years after the paper of Ross et al. [117]; combining gene expression array, chromatin-immunoprecipitation, and cell-based approach, Okamura et al. [90] demonstrated that Wnt/*β*-catenin induces the orphan nuclear receptor COUP-TFII [133] that recruits the silencing mediator of retinoid and thyroid hormone receptors (SMRT) co-repressor complex to the first intron of PPAR*γ* repressing its transcription [90]. Other regulatory proteins and networks, such as the Nemo-like kinase NLK [134], Smad/TFG*β* [135], the Wnt co-receptor antagonist Dickkopf-1 [119, 120], and TNF-*α* [136, 137], may also regulate PPAR*γ* interacting with Wnt.

PPAR*γ* agonists such as TZD, GW1929, or 15d-PGJ2 inhibit *β*-catenin signaling *in vitro* and *in vivo* [122–126]. In human myeloid and lymphoid leukemic cell lines PPAR*γ* activation induces cell-cycle arrest and apoptosis downregulating c-Myc through blockade of TCF activity [126]. In the colon PPAR*γ* activation suppresses epithelial cell turnover and *β*-catenin transcriptional activity retaining the *β*-catenin in the cytosol [123]. Conversely, the PPAR*γ* antagonist T0070907 promotes tumorigenesis in the small intestine and colon stimulating epithelial proliferation: this effect depends on increased expression of c-myc and Cyclin D1 genes and *β*-catenin activation [127, 138].

Several studies have demonstrated that PPAR*γ* directly interacts with *β*-catenin [128, 138, 139]. The functional interaction between *β*-catenin and PPAR*γ* requires the presence of PPAR*γ* co-receptor RXR [139] and involves the TCF/LEF binding domain of *β*-catenin and a catenin binding domain within PPAR*γ* [128]. It has been reported that the ligand-activated PPAR*γ* may retain *β*-catenin in the cytosol so reducing its transcriptional activity [138] and may determine a proteasomal degradation of the protein [125, 128]; however Lu and Carson [122] did not observe a reduction in *β*-catenin expression after TZD treatment *in vitro*.

Furthermore, PPAR*γ* may indirectly increase *β*-catenin degradation influencing the phosphorylation status of GSK3*β*. PPAR*γ* is a known activator of the phosphatase and tensin homologue deleted on chromosome 10 (PTEN), a phosphatase that inhibits AKT signaling [140]. Consequently, PPAR*γ* PTEN-mediated antagonism of AKT may reduce the inhibition of GSK3*β* enhancing proteasomal degradation of *β*-catenin, in agreement with other reports that have implicated PTEN as a negative regulator of *β*-catenin by way of AKT/GSK3*β* [141].

An axis PPAR*γ*-*β*-catenin-FOXO has been suggested by Almeida et al. during oxidative stress in the skeleton [118]. The authors first demonstrated that lipid oxidation, that causes oxidative stress, and PPAR*γ* expression increase with aging; the authors then go on demonstrating that ROS activate FOXO that decrease Wnt/*β*-catenin by competition; in turn, the ensuing PPAR*γ* increased expression and activation further sink *β*-catenin signaling [118]. However Almeida et al. [118] did not specify the PPAR*γ*-FOXO relationship.

FOXO may modulate PPAR*γ* at the mRNA and protein levels [129–131]. In fact, FOXO1 acts as a transcriptional repressor binding to the PPAR*γ* promoter [130, 131] and may reduce PPAR*γ* activity through a transrepression mechanism that involves a direct protein-protein interaction [129, 131]. The transrepression mechanism is dependent on PPAR*γ* activation by its ligands and is mediated by a 31-amino-acid domain within FOXO1 that contains one inverted and two atypical LXXLL motifs [131]. Likewise it has been reported that in 293T cells cotransfection with increasing amounts of expression vectors encoding PPAR*γ* and RXR results in a dose-dependent inhibition of FOXO1-driven reporter activation [129], creating a regulatory feedback loop PPAR*γ*-FOXO; however it is not clear if the reported effect is due to a transrepression mechanism or is an indirect effect of PPAR*γ* : RXR dimers.

It has been suggested that PPAR*γ* may regulate FOXO1 through modulation of AKT with a mechanism that may explain the signal cascade of insulin [131]; this regulatory circuit could however also act during oxidative stress. As mentioned before, FOXO proteins are negatively regulated by PI3k-AKT-mediated phosphorylation; consequently, PPAR*γ*, inducing PTEN, may block AKT activation and hence may, indirectly, increase FOXO activity. Interestingly, Sakamoto et al. [142] demonstrated in PTEN-deficient Jurkat cells an AKT- and NRF2-dependent enhanced activation of ARE containing genes; this finding suggests that the regulatory network PPAR*γ*-PTEN-AKT that acts on FOXO with activating effects may also act on NRF2; however given that AKT increases NRF2 activity [75], the axis PPAR*γ*-PTEN-AKT, conversely to the effect on FOXO, should reduce NRF2-induced transcription.

Moreover, PPAR*γ* may influence FOXO acting on p38-MAPK, SIRT histone deacetylase, and the NF-*κ*B. NF-*κ*B and p38 inactivate FOXO and increase their ubiquitination-mediated degradation [111, 113, 114]. PPAR*γ* may counteract the effects of both these pathways: in fact PPAR*γ* transrepresses (and it is repressed by) NF-*κ*B and influences the p38 MAPK signaling cascade increasing the expression of heat shock protein 27 (Hsp27) [143]. The Hsp27, in turn, possesses antioxidant properties [144] and might act indirectly on FOXO with both positive and negative effects (inhibiting NF-*κ*B [145] and activating AKT [146], resp.). This latter effect may also explain the previously discussed finding of a PPAR*γ*-dependent inhibition of FOXO1 [129].

Acetylation/deacetylation regulates different functions of FOXO proteins. It has been demonstrated that the deacetylase SIRT1 deacetylates FOXO proteins in response to stress [147, 148], increases the ability of FOXO to induce cell-cycle arrest, but diverts FOXO-dependent response from apoptosis [147]; interestingly in the heart, whereas moderate levels of SIRT protect from oxidative stress, high levels of the deacetylase may increase the oxidative stress and apoptosis [148]. SIRT2, that shares similar function with SIRT1, suppresses adipogenesis by deacetylating FOXO1 to promote FOXO1's binding to PPAR*γ* and subsequent repression on PPAR*γ* transcriptional activity [132]. Furthermore, SIRT1 represses PPAR*γ* by docking with its cofactors nuclear receptor co-repressor (NCoR) and SMRT [149]. Finally, a negative feedback and self-regulating loop links SIRT1 to PPAR*γ*: Han et al. [150] demonstrated that both PPAR*γ* and SIRT1 bind the SIRT1 promoter inhibiting the transcription of the deacetylase; moreover, PPAR*γ* directly interacts with SIRT1 and inhibits SIRT1 activity; the association SIRT1-PPAR*γ* appears to be mediated by the acetylation status of PPAR*γ* [150]. Acetylation of nuclear receptor is usually associated with an increase in the transcriptional activity; although there is still no a direct evidence on PPAR*γ*, it has been demonstrated that during senescence both acetylation and activity of PPAR*γ* increase [150, 151] suggesting that also in the case of PPAR*γ* acetylation might increase the activity. Interestingly, the increased acetylation of PPAR*γ* may determine an inhibition of SIRT1 via direct binding, thus limiting the activating deacetylation of FOXO. Furthermore the inhibition of SIRT PPAR*γ*-mediated may also explain the finding that PPAR*γ* is involved in the senescence [152]. Paradoxically, inhibition of SIRT1 may increase the risk of cell death directing the FOXO-dependent response toward apoptosis [147].

## 3. Conclusions

A prolonged condition of oxidative stress, as a result of imbalance between antithetic prooxidation and anti-oxidation forces, may lead the cell to her apoptotic or necrotic doom.

In this delicate and intricate game of equilibrium, when the cell may survive or die depending on her ability to sustain and repair the damage, PPAR*γ* stands out as a central player.

Given that natural ligands of PPAR*γ* are produced during oxidative stress, PPAR*γ*, if already expressed, may be one of the first responders directly inducing an arsenal of antioxidant molecules, inhibiting prooxidants and in the same time protecting the cells from apoptosis.

**Figure 2:**
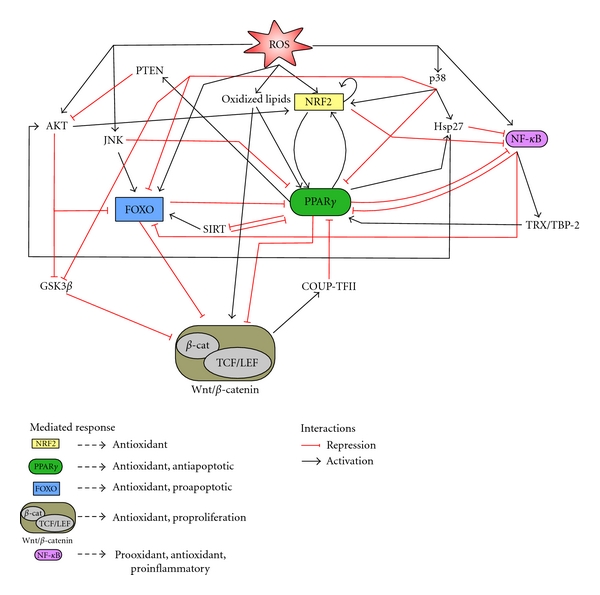
Crosstalk of PPAR*γ* with NRF2, Wnt/*β*-catenin, and FOXO signaling pathways in oxidative stress response. ROS and other reactive species activate NRF2 and PPAR*γ* that are linked by a positive feedback loop that sustains their expression. Through a negative feedback PPAR*γ* inhibits Wnt/*β*-catenin and induces cell block. Activation of FOXO in turn blocks Wnt/*β*-catenin (diverting *β*-catenin from proliferation to resistance and apoptosis) and PPAR*γ*. Finally, the result of these interactions, survival or cell death, depends on the action of other signaling pathways and regulatory circuits.

It is obvious that in this defense mechanism PPAR*γ* does not act alone. Indeed the nuclear receptor is at the point of interconnection of various pathways, specifically the NRF2 and the Wnt/*β*-catenin and FOXO pathways (Figure 2).

The redox-sensitive nuclear factor NRF2 plays vital role in cytoprotection against oxidative and electrophilic stress and is induced by oxidative stress. The pieces of evidence recollected in this paper demonstrate that PPAR*γ* and NRF2 are linked by a positive feedback loop that sustains the expression of both transcription factors and of antioxidant and prosurvival genes, as long as oxidative stress goes on. At the same time, the two genes exert a potent anti-inflammatory action inhibiting the NF-*κ*B pathway.

Conversely, PPAR*γ* and the proproliferative Wnt/*β*-catenin pathway are associated by a negative feedback loop. The role of Wnt/*β*-catenin in response to oxidative stress is not straightforward: it has been demonstrated that the pathway is able to positively regulate the expression of some antioxidant genes and the absence of *β*-catenin increases the oxidative stress; however, in light of the interaction of Wnt/*β*-catenin with PPAR*γ*, the inhibition of Wnt/*β*-catenin pathway may be a necessary step to block cell proliferation and to mount an antioxidant response inducing the expression of the anti-inflammatory and antioxidant PPAR*γ*. Furthermore the block of Wnt/*β*-catenin pathway and the diversion of *β*-catenin from the cognate TCF towards the FOXO transcription factors increase not only oxidative stress response but also the rate of apoptosis.

PPAR*γ* interacts also with FOXO. The two transcription factors elicit a positive response to oxidative stress but FOXO, binding with *β*-catenin, may induce apoptosis whereas PPAR*γ* increases the expression of the antiapoptotic Bcl-2. The relation PPAR*γ*-FOXO is ambivalent: FOXO directly inhibits PPAR*γ* and indirectly induces it, sinking the negative effect of Wnt/*β*-catenin; on the other hand PPAR*γ* acting exclusively indirectly is both inducer and repressor. This complicated relationship probably reflects the different effect on apoptosis, clearly proapoptotic FOXO, mostly antiapoptotic PPAR*γ*. PPAR*γ* may retard apoptosis inducing Bcl-2 and inhibiting FOXO; however this mechanism is self-limiting because activating FOXO, PPAR*γ* creates a negative loop that may increase the chances of cellular death.

Interestingly PPAR*γ* may be the bridge linking NRF2 to Wnt/*β*-catenin and FOXO pathways. Activating PPAR*γ*, NRF2 not only potentiate the cellular oxidative response but may also overcome the negative feedback loop PPAR*γ*-Wnt/*β*-catenin inducing cell cycle arrest giving more time to the cells to repair the damage; at the same time, through PPAR*γ*, NRF2 may fine-tune the stress resistance and apoptotic FOXO-mediated responses to oxidative stress. However, because of the multiple regulatory links we have described, this interaction goes in both ways giving that Wnt/*β*-catenin and FOXO, inhibiting PPAR*γ*, may influence NRF2 activity.

Although the experimental pieces of evidence recollected in this paper have been obtained from various experimental models and conditions (see Tables 1 and 2) and need further verifications, they clearly suggest that the role of PPAR*γ* in oxidative stress is essentially devoted to the quenching and containment of the damage and to foster cell survival; furthermore they depict a new signaling network NRF2-PPAR*γ*-Wnt/*β*-catenin-FOXO that may be exploited for the treatment of oxidative-related diseases.

In conclusion, during oxidative stress PPAR*γ* promotes an antioxidant response integrating NRF2, Wnt/*β*-catenin, and FOXO pathways, but clearly the end result of oxidative stress, survival or apoptotic death, does not depend only on these sole genes but also on the net effect of other signaling pathways and regulatory circuits that converge on, and interact with, these transcription factors.

## Figures and Tables

**Table 1:** Experimental data for PPAR*γ*-modulated genes during oxidative stress.

Target	Effect	Tissue or cell type	Type of evidence	References
Bcl-2	↑ expression	Cardiomyocyte and glial cells	*In vitro*	[47, 49]
Catalase	↑ expression	Adipocytes, microvascular endothelial cells	*In vitro*	[45, 46]
CD36	↑ expression	Macrophages	*In vitro*	[53, 54]
COX-2	↑↓ expression	Hepatocytes, neurons, pancreatic islets, macrophages, astrocytes	*In vitro* *In vivo*	[29, 41, 57–59, 63]
eNOS	↑ expression	Endothelial cell	*In vivo*	[33]
GPx3	↑ expression	Skeletal muscle cells	*In vitro*	[52]
HO-1	↑↓ expression	Hepatocytes, human vascular cells, retina, hippocampal neurons,	*In vitro* *In vivo*	[41, 50, 55, 56]
iNOS	↓ expression	Neurons, macrophages, pancreatic islets, Schwann cells	*In vivo* *In vitro*	[29, 57–59, 62]
MnSOD	↑ expression ↑ activity	Hippocampal neurons, heart	*In vivo*	[50, 51]
UCP2	↑ expression	Sympathetic premotor neurons, liver	*In vivo*	[35, 38]

↑ = increasing; ↓ = decreasing; ↑↓= increasing and decreasing effects described.

**Table 2:** Main experimental data linking PPAR*γ* to NRF2, Wnt/*β*-catenin, and FOXO pathways.

Interaction	Gene	Target	Effect	Tissue or cell type	Type of evidence	References
NRF2-PPAR*γ*	NRF2	PPAR*γ*	↑ expression	Liver, lung	*In vitro* *In vivo*	[79–81]
	PPAR*γ*	NRF2	↑ expression	Lung, hepatocytes, macrophages, vascular tissue	*In vitro* *In vivo*	[73, 77, 80, 82]
Wnt/*β*-catenin-PPAR*γ*	Wnt/*β*-catenin	PPAR*γ*	↓expression	Pre-adipocytes, adipocytes, skeleton, hepatic stellate cells, neurons, cancer cells	*In vitro* *In vivo*	[6, 90, 117–121]
	PPAR*γ*	Wnt/*β*-catenin	↓transcriptional activity ↑ proteasomal degradation ↑ cytoplasmic localization	Colon, small intestine, colon cancer cells, hepatocytes, myeloid and lymphoid leukemic cells	*In vitro* *In vivo*	[122–128]
FOXO-PPAR*γ*	FOXO	PPAR*γ*	↓ expression ↓ activity	Adipocytes	*In vitro* *In vivo*	[129–132]
	PPAR*γ*	FOXO	↓ activity	Adipocytes	*In vitro*	[129]

↑ = increasing; ↓ = decreasing.

## Abbreviations

15d-PGJ2: 15-Deoxy-delta (12, 14)-prostaglandin J(02)
APC: Adenomatous polyposis coli
ARE: Antioxidant response element
Bcl-2: B-cell lymphoma 2
CYP: Cytochrome P450
DEN: Diethylnitrosamine
DR-1: Direct repeat 1
DR-2: Direct repeat 2
ERK: Extracellular-signal-regulated kinase
FOXO: Forkhead box proteins O
GSH: Glutathione
GSK3*β*: Glycogen synthase kinase 3*β*
GST: Glutathione S-transferase
HDAC3: Histone deacetylase 3
Hsp27: Heat shock protein 27
IGF-1: Insulin-like growth factor 1
IKK*β*: Inhibitor of NF-*κ*B subunit *β*
JNK: c-Jun N-terminal kinases
Keap1: Kelch-like ES-associated protein 1
LPS: Lipopolysaccharide
LRP5/6: Low-density lipoprotein receptor-related protein 5/6
MAPK(s): Mitogen-activated protein kinase(s)
MnSOD: Manganese SOD
NAFLD: Nonalcoholic fatty liver disease
NASH: Nonalcoholic steatohepatitis
NcoR: Nuclear receptor co-repressor
NF-*κ*B: Nuclear factor kappa-light-chain-enhancer of activated B cells
NRF2: Nuclear factor erythroid 2-related factor 2
PGH2: Prostaglandin H2
PKC: Protein kinase C
PPAR: Peroxisome-proliferator activator receptors
PPRE: PPAR response element
PTEN: Phosphatase and tensin homologue deleted on chromosome 10
ROS: Reactive oxygen species
RXR: Retinoid X receptor
SMRT: Silencing mediator of retinoid and thyroid hormone receptors
SOD: Superoxide dismutase
TBP-2: TRX-binding protein-2
TCF/LEF: DNA binding protein T-cell factor/Lymphoid enhancer factor
TRX: Thioredoxin
TZD: Thiazolidinediones
UCP2: Mitochondrial uncoupling protein 2.
